# Analysis of the spatial distribution of *Aedes albopictus* in an urban area of Shanghai, China

**DOI:** 10.1186/s13071-021-05022-8

**Published:** 2021-09-26

**Authors:** Yibin Zhou, Hongxia Liu, Peien Leng, Jiang Zhu, Shenjun Yao, Yiyi Zhu, Huanyu Wu

**Affiliations:** 1 Department of Infectious Disease Control, Shanghai Municipal Center for Disease Control and Prevention, Shanghai, 200336 China; 2 Key Laboratory of Geographic Information Science (Ministry of Education), East China Normal University, Shanghai, 200241 China

**Keywords:** *Aedes albopictus*, Mosquito oviposition trap, Binomial areal kriging model, Spatial distribution

## Abstract

**Background:**

*Aedes albopictus* is a vector of major arboviral diseases and a primary pest in tropical and temperate regions of China. In most cities of China, the current monitoring system for the spread of *Ae. albopictus* is based on the subdistrict scale and does not consider spatial distribution for analysis of species density. Thus, the system is not sufficiently accurate for epidemic investigations, especially in large cities.

**Methods:**

This study used an improved surveillance program, with the mosquito oviposition trap (MOT) method, integrating the actual monitoring locations to investigate the temporal and spatial distribution of *Ae. albopictus* abundance in an urban area of Shanghai, China from 2018 to 2019. A total of 133 monitoring units were selected for surveillance of *Ae. albopictus* density in the study area, which was composed of 14 subdistricts. The vector abundance and spatial structure of *Ae. albopictus* were predicted using a binomial areal kriging model based on eight MOTs in each unit. Results were compared to the light trap (LT) method of the traditional monitoring scheme.

**Results:**

A total of 8,192 MOTs were placed in the study area in 2018, and 7917 (96.6%) were retrieved, with a positive rate of 6.45%. In 2019, 22,715 (97.0%) of 23,408 MOTs were recovered, with a positive rate of 5.44%. Using the LT method, 273 (93.5%) and 312 (94.5%) adult female *Ae. albopictus* were gathered in 2018 and 2019, respectively. The *Ae. albopictus* populations increased slowly from May, reached a peak in July, and declined gradually from September. The MOT positivity index (MPI) showed significant positive spatial autocorrelation across the study area, whereas LT collections indicated a nonsignificant spatial autocorrelation. The MPI was suitable for spatial interpolation using the binomial areal kriging model and showed different hot spots in different years.

**Conclusions:**

The improved surveillance system integrated with a geographical information system (GIS) can improve our understanding of the spatial and temporal distribution of *Ae. albopictus* in urban areas and provide a practical method for decision-makers to implement vector control and mosquito management.

**Graphical abstract:**

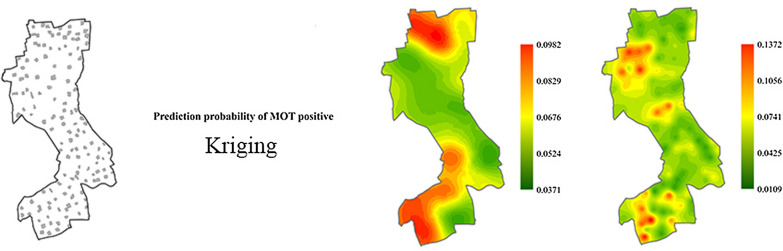

## Background

*Aedes albopictus*, also known as the Asian tiger mosquito, has invaded all continents except Antarctica during the last 30–40 years [1, 2]. *Aedes albopictus* is a primary human-biting pest species that significantly reduces the quality of life of infected persons, and is an invasive vector of major arboviral diseases, such as dengue, chikungunya, yellow fever, and Zika. In China, *Ae. albopictus* has adapted to low temperatures and is currently a primary nuisance pest and disease vector in the tropical and temperate regions of the country [3]. This species is present in regions where *Aedes aegypti* (Linnaeus) is absent [4], including Shanghai, and dengue is one of the most widely transmitted diseases carried by *Ae. albopictus* in China [5]. *Aedes albopictus* was reported to be the primary vector of several epidemics in Guangzhou Province (37,354 laboratory-confirmed cases) in 2014 [6] and Zhejiang Province (adjacent to Shanghai) in 2004 [7], 2009 [8], and 2017 [9].

The dengue case reported in 2017 was the first autochthonous dengue case in Shanghai in the last five decades [10]. Since then, three cases have been reported [11], and *Ae. albopictus* has been at the top of the list for vector control and surveillance in Shanghai. The public health service developed a monitoring system for *Ae. albopictus* to obtain information regarding its temporal evolution using the light trap (LT) and mosquito oviposition trap (MOT) methods in 2010. This system is used for surveillance of the *Ae. albopictus* population and biting rates based on data collected from subdistricts. However, a disadvantage of the surveillance network is that it does not integrate geographical information, and the subdistrict scale, which is usually > 3 km, is large. As a result, only the regional average density of *Ae. albopictus* can be obtained with this monitoring system.

Geostatistical methods, which integrate the actual locations of samples, have been used to investigate the spatial distribution of mosquitoes [12] and several mosquito-transmitted diseases, including malaria [13, 14] and dengue fever [15, 16]. Global and local indicators of spatial autocorrelation, such as Moran’s *I* [17] or local indicators of spatial association (LISA) [18], have been applied to study pests, including mosquitoes [19, 20]. These indicators can detect hot spots of mosquito abundance and predict the significance of clustering and the effect of disease control [21]. Among the geostatistical methods, kriging interpolation [22] can predict the vector abundance in unsampled areas. Albieri et al. used kriging interpolation to predict the mosquito population distribution at the provincial and municipal scales in northern Italy [23], and Azil et al. used kriging to analyze the costs of dengue vector surveillance and control programs in Australia [24]. In addition, Giordano et al. created a more efficient larvicide control program for West Nile virus awareness campaigns in Canada by using kriging interpolation [25].

In China, the density of *Ae. albopictus* is usually assessed based on the *Aedes*-positive rate in MOT monitoring. MOT is a standard method of surveillance for the temporal and spatial distributions of container-inhabiting mosquitoes, including *Ae*. *albopictus* [26]. Current *Aedes* adult sampling methods, such as LTs, are labor-intensive, expensive, and challenging to implement in large numbers [27]. As an alternative method, MOTs are artificial traps for *Aedes* egg collection. Compared to traditional traps, MOTs are more convenient and inexpensive to implement because they are easy to carry and do not require electricity or bait supplies. This method can detect the presence of gravid *Aedes* females with higher sensitivity, especially at low densities [28]. However, there have been rare reports [29] on the spatial interpolation of positive rates of *Ae. albopictus*.

The present study set out to evaluate the temporal and spatial distributions of *Ae. albopictus* using the MOT method, investigate the autocorrelation of *Ae. albopictus* abundance, and estimate *Ae. albopictus* abundance at non-sampling locations using the binomial areal kriging model [30] in an urban area of Shanghai, and identify hot spots and risk areas of high infestation. We created an improved surveillance program, which included the location of the traps and a change in the scale from 14 subdistricts to 133 monitoring units, for *Ae. albopictus* in an urban area of Shanghai from 2018 to 2019. Eight MOTs were applied in each unit to predict *Ae. albopictus* abundance and spatial structure at non-sampling locations. The LT method from the original monitoring scheme was also used and compared to the improved MOT method.

## Methods

### Selection of the study area

**Fig. 1:**
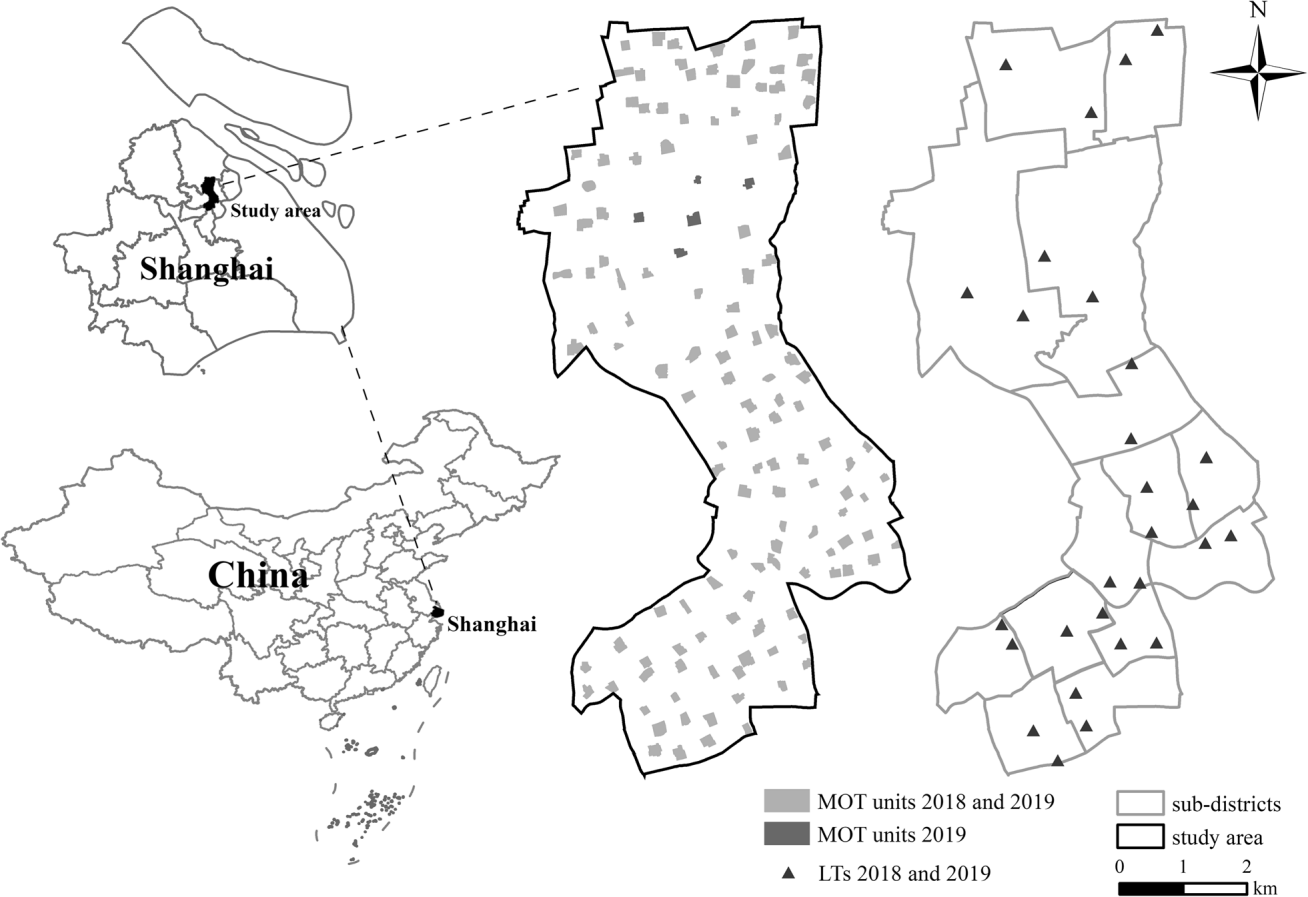
Map of Shanghai, China, and locations of MOT monitoring units and LTs in the study area. *MOT* mosquito oviposition trap, *LT *light trap

Shanghai is situated at 31°12′N latitude and 121°30′E longitude in the eastern part of the alluvial plain of the Yangtze Delta, adjacent to the Yangtze River estuarine and the East China Sea. It has four distinct seasons (spring from March to May, summer from June to August, autumn from September to November, and winter from December to February) and abundant precipitation, with a subtropical monsoon climate. The mean annual temperature in Shanghai is ~17 °C, and the mean annual precipitation is greater than 1100 mm, with 53% occurring between June and September. The study area is situated in the center of Shanghai, China, with a total area of 37.37 km^2^, measuring 6.15 km from east to west and 11.93 km from south to north (Fig. 1). In 2018, the study region included 14 subdistricts with a resident population of 1,057,700 [31].

### Meteorological data

The monthly total precipitation and monthly mean maximum and minimum temperatures were calculated based on data from the China Meteorological Administration [32]. Weather variables were recorded at Xujiahui, which is located ~2 km from the study area.

### Entomological survey

The abundance of *Ae. albopictus* was analyzed using the MOT (Tian^®^, Kaiqi Co., Ltd., Shanghai, China) and LT methods (Tian^®^, Kaiqi Co., Ltd., Shanghai, China). The MOTs provide artificial breeding sites for container-breeding mosquitoes, including *Ae. albopictus*, and positive MOTs were defined as those containing adults or eggs of *Ae. albopictus*. The LTs are baited with carbon dioxide to attract adult mosquitos, and the number of female adult mosquitoes captured was used as the index.

Each MOT [33] consists of a transparent cylindrical plastic jar (100 mm high, 70 mm diameter, 66 mm internal diameter) with a concave bottom (20 mm inward) and a black top cover with three conical openings 100 mm in diameter. When used as a collection container, white filter paper 70 mm in diameter, which is used as an egg deposition substrate, was placed inside the bottom of the MOT and 25 ml of dechlorinated water poured into the jar to keep the paper moist but not submerged. MOTs were placed outdoors on grasslands, and kept away from direct sunlight, rain, and wind, at ground level by a skilled technician. To reduce competition among the MOTs, all of the traps were separated by a distance of more than 20 m and maintained unchanged until the end of the study.

In 2018, the MOTs were placed once a month between April and November. To enhance the data during the peak period, the frequency was increased in 2019 to once a week in week 20, week 23, week 25, weeks 27–39, and weeks 41–46. The MOTs that were removed, emptied, or interfered with for any reason were excluded from further analysis. After 4 days, each MOT was collected and species identification performed in the laboratory under a stereomicroscope. The MOT positivity index (MPI) was calculated as follows: MPI = number of *Aedes*-positive MOTs / total number of MOTs retrieved × 100%.

The study area had 14 subdistricts containing 276 communities. Every two adjacent communities in one subdistrict were grouped into one polygon. When the number of communities in one subdistrict was odd, there was a polygon composed of three communities. We found a residential area with vegetative coverage in each polygon as the monitoring unit for the MOT (Fig. 1) and decided to place eight MOTs in each unit. The reasoning was as follows. First, the smallest subdistrict had six monitoring units. Second, this ensured that there were nearly 50 MOTs in each subdistrict, as noted in the Implementation Program of National Vector Surveillance launched by China’s Center for Disease Control and Prevention. Third, the number of MOTs in each monitoring unit was consistent. In 2018, the center of the study area was under construction and MOTs could not be placed, so we put 128 units in the rest of the area (Fig. 1). The MPI values of the eight MOTs were used to represent the *Ae. albopictus* density of the unit. In 2019, we added five monitoring units in the middle of the study area, which led to a total of 133 monitoring units (Fig. 1).

Due to the low density of *Ae. albopictus* in April and the lower sensitivity of the LT compared with the MOT, the LT could not catch adult mosquitoes. Thus, on the third Wednesday of each month from May to November, the Center for Disease Control and Prevention set two LTs in every subdistrict throughout Shanghai as part of a citywide mosquito surveillance program. LTs were usually collected from 4 to 10 pm. The contents of these traps were sent to the laboratory for species identification, and only female *Ae. albopictus* were collected for data analyses.

The georeferenced positions of the MOT monitoring units and LTs in 2018 and 2019 are presented in Fig. 1.

### Cluster analysis

The monitoring data were assigned to groups based on the type of trap and year of collection (i.e., MOT2018, MOT2019, LT2018, and LT2019) and analyzed. The total yearly collection of each LT and the mean MPI of each unit were calculated. Geostatistical analyses were conducted using the ArcGIS software (version 10.8; ESRI, Beijing, China) Spatial Statistics toolbox and data imported from Microsoft Excel 2019. Near table analyses were performed using the Generate Near Table tool to calculate the distance from each feature to its nearest neighboring feature based on the Euclidean distance.

We evaluated whether the mosquito abundances were spatially autocorrelated by calculating the incremental spatial autocorrelation (global Moran’s *I* at multiple distances). The global Moran’s *I* [17] was tested using the permutation procedure based on feature locations and attribute values (yearly total collection from each LT and mean MPI of each unit) against the null hypothesis (the absence of spatial autocorrelation). This analysis identified the spatial patterns in the study area but did not indicate where such clusters occurred, which was determined by the local Moran’s *I* [18]. The local Moran's *I* analysis was performed to assess the presence of hot spots with significant clusters, cold spots, and spatial outliers. For polygon MOT monitoring units, feature centroids were used in distance computations.

### Geostatistical analysis

The kriging interpolation method [22] was used to quantify the spatial structure of the data and predict species abundance at unsampled locations. The binomial areal kriging model [30] is a geostatistical interpolation technique that extends the kriging theory to count data over polygons. In 1991, McNeill [34] derived the formulas for binomial kriging models. This step was performed using the ArcGIS 10.8 Geostatistical Analyst extension, including exploratory statistical analysis and variogram modeling. Each polygon of the input data contained a count (number of positive MOTs) and a population value (number of MOTs retrieved). The output was a figure predicting the positive rate and its standard error at each location.

A leave-one-out cross-validation method was used to determine whether the kriging interpolation provided reliable estimates at unsampled locations. The criteria used for accurate prediction in the cross-validation were as follows: root mean square standardized (RMSS) ~1, mean standardized (MS) ~0, and root mean square (RMS) approximately the average standard error (ASE).

### Digital map

Digital maps of China and Shanghai from the National Catalogue Service for Geographic Information (National Bureau of Surveying & Mapping, P.R. China), available at a scale of 1:1,000,000, were used as background for mapping. The shapefile of MOT monitoring units was produced by hand digitization of an aerial picture (Google Earth, May 2018; May 2019) in ArcGIS 10.8. The locations of LTs were georeferenced using GPS CHCNAV X360H (WGS 1984 coordinate system) and later projected onto the UTM projection at the 51 N zone.

## Results

### Mosquito collection

In 2018, a total of 8192 MOTs were placed in the study area, and 7917 (96.6%) of them were retrieved, with a positive rate of 6.45%. In 2019, 22,715 (97.0%) of 23,408 MOTs were recovered, with a positive rate of 5.44%. LTs collected both male and female adult *Ae. albopictus*, with females constituting the majority; 273 (93.5%) and 312 (94.5%) adult female *Ae. albopictus* were gathered in 2018 and 2019, respectively.

### Monthly distribution of *Ae. albopictus*

**Fig. 2:**
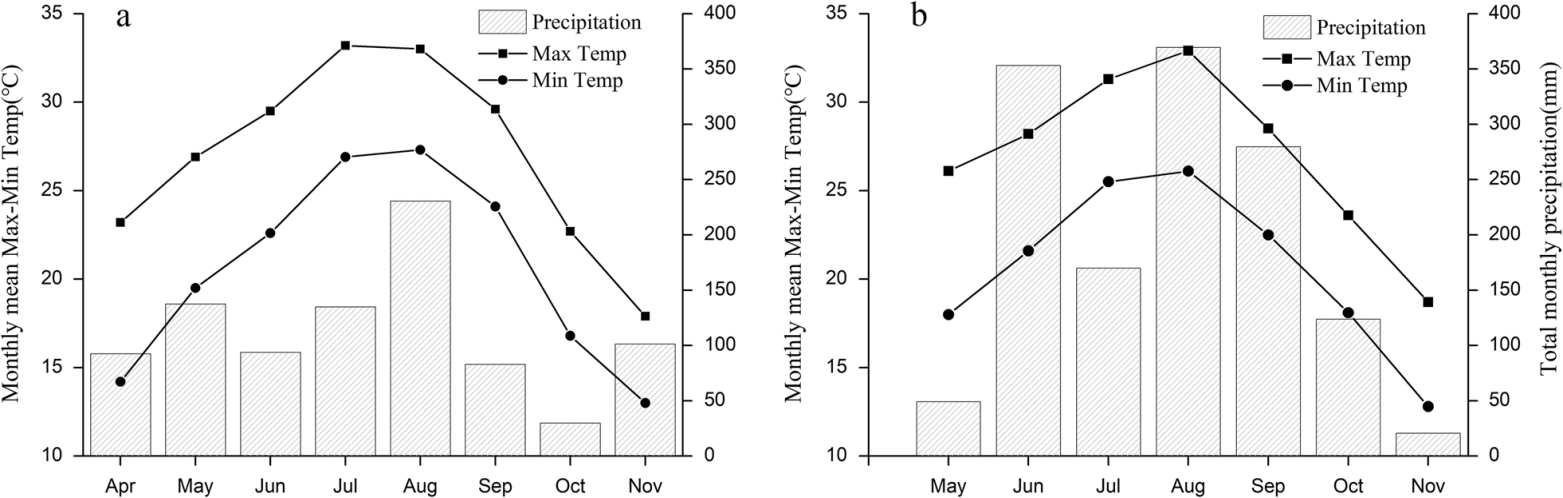
Monthly maximum–minimum temperature and precipitation: **a** 2018, **b** 2019

The monthly mean temperature reached a peak in July (33.2 °C) in 2018 and in August (32.9 °C) in 2019. The monthly precipitation was highest in August in both 2018 (230.5 mm) and 2019 (369.5 mm; Fig. 2). The total precipitation from May to October was almost twice as high in 2019 (1345.8 mm) as in 2018 (709.7 mm). In addition, the mean monthly maximum temperature from July to September was higher in 2018 (31.9 °C, Fig. 2a) than in 2019 (30.9 °C, Fig. 2b).

**Fig. 3:**
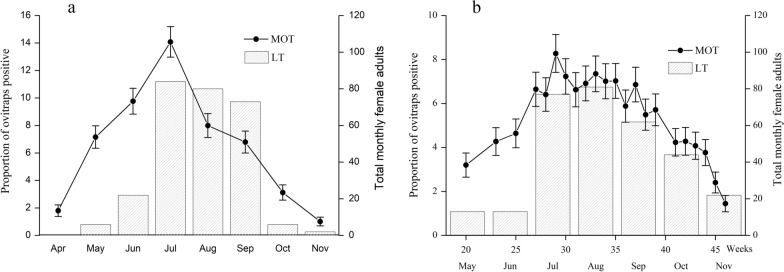
The monthly MPI and number of female adult *Ae. albopictus* captured by LTs: **a** 2018, **b** 2019. *MPI* mosquito oviposition trap positivity index, *LT* light trap

As shown in Fig. 3, lower levels of oviposition were detected by MOTs in April 2018 and May 2019, with both MPIs peaking in July. In 2018, the monthly MPI peaked at 14.08%, whereas in 2019 it peaked at 8.28% in the 29th week (July). The MPI peak during the 2018 mosquito season was higher than the peak in the 2019 season. There was also a significant peak in the number of female adults collected by LTs from July to September in both years.

In 2018, the number of adult female *Ae. albopictus* collected by LTs peaked in July, which is consistent with the MPI. The number of female adults collected by LTs increased from May to July, reaching the highest values in July and August, and then decreasing in September. In general, the seasonal fluctuation curves in 2018 and 2019 indicated that *Ae. albopictus* populations in the urban area of Shanghai slowly increased from May, peaked in July, and declined gradually from September to October. The Spearman correlation coefficient (*r*) between the monthly number of adult female *Ae. albopictus* collected by LTs and monthly MPI was 0.792 (*P* = 0.033 4, *df* = 6) in 2018 and 0.756 (*P* = 0.048 9, *df* = 6) in 2019.

### Spatial distribution of MOTs and LTs

The near table analyses produced the mean, minimum, and maximum distances between traps (Table 1). The mean distance between MOT monitoring units was 197.77 m in 2018, 188.57 m in 2019, and 702.05 m between LTs in both 2018 and 2019.

**Table 1:** Results of the near table

Trap collection	MOT2018	MOT2019	LT2018 and 2019
Numbers of units	128	133	28
Minimum distance (m) to the nearest neighbor	8.62	8.62	380.08
Maximum distance (m) to the nearest neighbor	439.33	504.35	1538.84
Mean distance (m)	179.77 ± 98.87	188.57 ± 107.25	702.05 ± 263.50

*MOT* mosquito oviposition trap, *LT* light trap

### Cluster analysis of *Ae. albopictus* abundance

We performed incremental spatial autocorrelation analyses to evaluate the spatial autocorrelation of *Ae. albopictus* abundance across the study area. Only the MOT method demonstrated a significant positive spatial autocorrelation based on the mean MPI of each unit, which peaked at 651 m in 2018 and 528 m in 2019. The LT method did not show a significant spatial autocorrelation of *Ae. albopictus* abundance based on the total collected in each LT (Table 2).

**Table 2:** Results of incremental spatial autocorrelation analysis

Trap collection	MOT2018	MOT2019	LT2018	LT2019
Number of units	128	133	28	28
The distance of peak global Moran’s *I* (m)	651*	528*	680*	716*
Global Moran’s *I*	0.297	0.279	0.227	0.303
*Z*-score	4.542	3.247	1.050	1.480
*P*-value	< 0.001**	< 0.001**	0.293***	0.139***

*MOT* mosquito oviposition trap, *LT *light trap

*Some units with no neighbors at this distance

**Significant positive spatial autocorrelation when *P* < 0.05

***Random patterns when *P* > 0.05

**Fig. 4:**
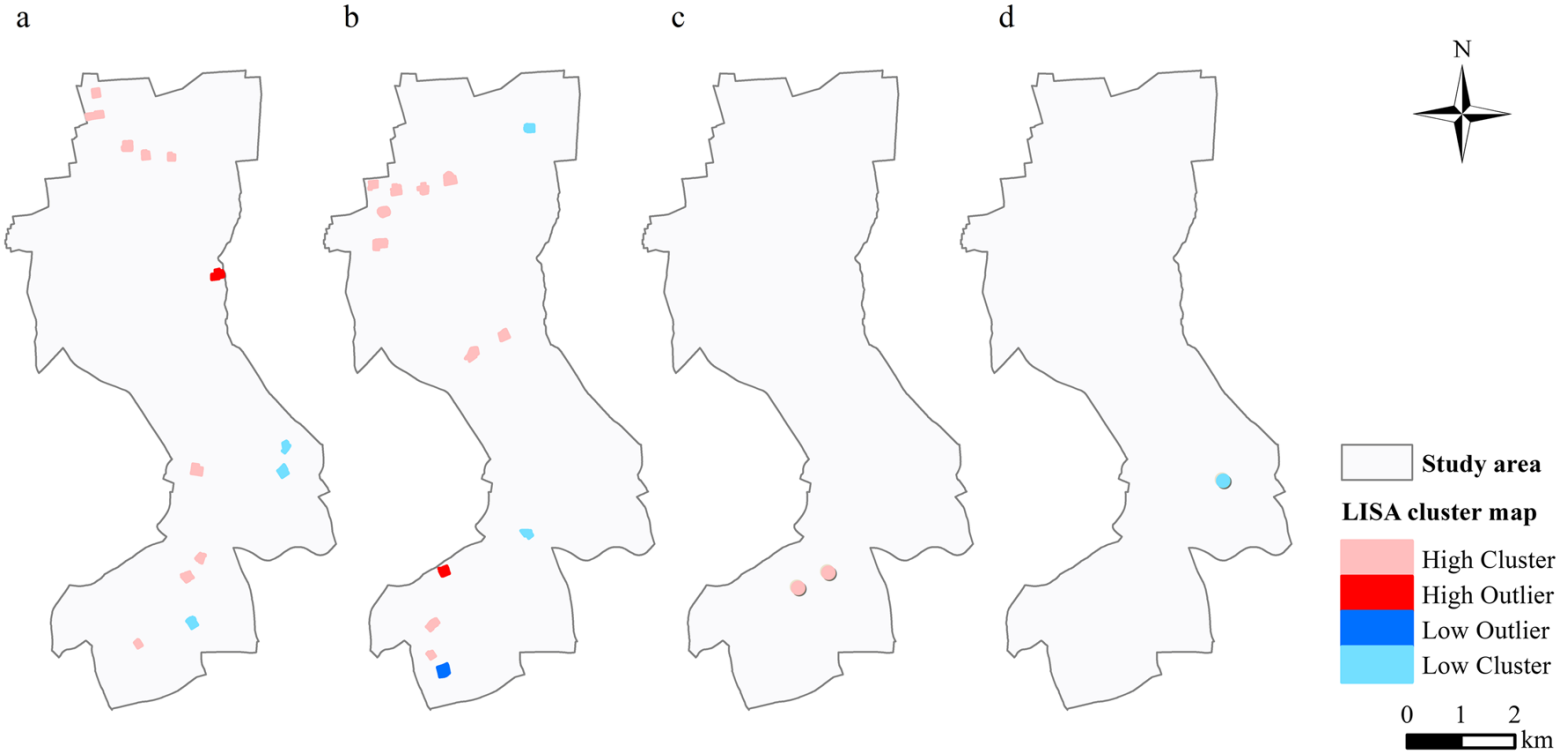
Local Moran’s *I* maps: **a** MOT2018, **b** MOT2019, **c** LT2018, **d** LT2019

Local Moran’s *I* was used to determine the locations of hot spots, cold spots, and spatial outliers. MOT2018, MOT2019, LT2018, and LT2019 had 13, 14, 2, and 1 clusters or outliers, respectively (Fig. 4).

### Prediction of *Ae. albopictus* abundance at non-sampling locations

**Fig. 5:**
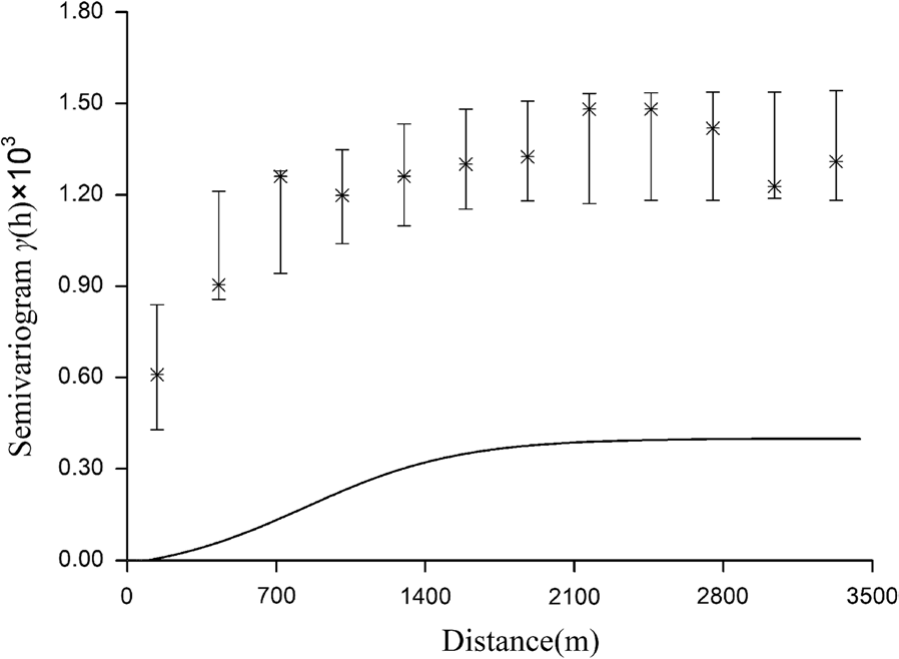
Deconvoluted point semivariogram (line) and re-estimated empirical semivariogram values for polygons (stars) and their 90% confidence intervals (vertical lines) for MOT2018

**Fig. 6:**
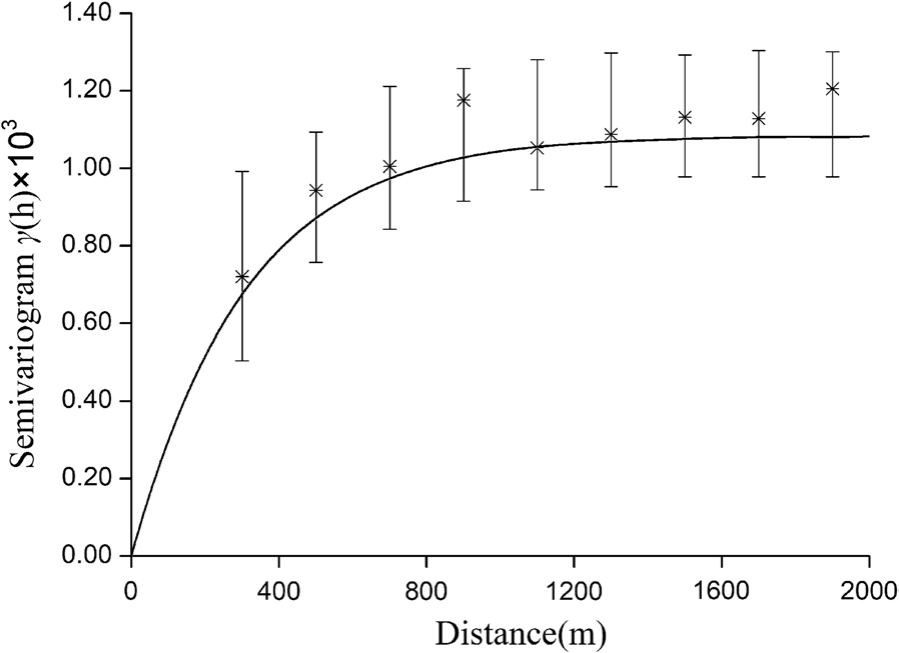
Deconvoluted point semivariogram (line) and re-estimated empirical semivariogram values for polygons (stars) and their 90% confidence intervals (vertical lines) for MOT2018

We found that the mean MPI of each unit was suitable for kriging interpolation because it demonstrated significant positive spatial autocorrelation. There was no spatial autocorrelation for LT2018 and LT2019, and spatial interpolation was not permitted. We constructed the binomial areal kriging model using the yearly number of positive MOTs and the number of MOTs retrieved in each unit. Semivariograms created with the binomial areal kriging model showed spatial dependence (range) within ~1900 m and 900 m for MOT2018 and MOT2019 (Figs. 5, 6), respectively, beyond which the semivariance remained constant. The best-fitting model for 2018 was the Gaussian model, and the exponential model best fit the data for 2019.

**Fig. 7:**
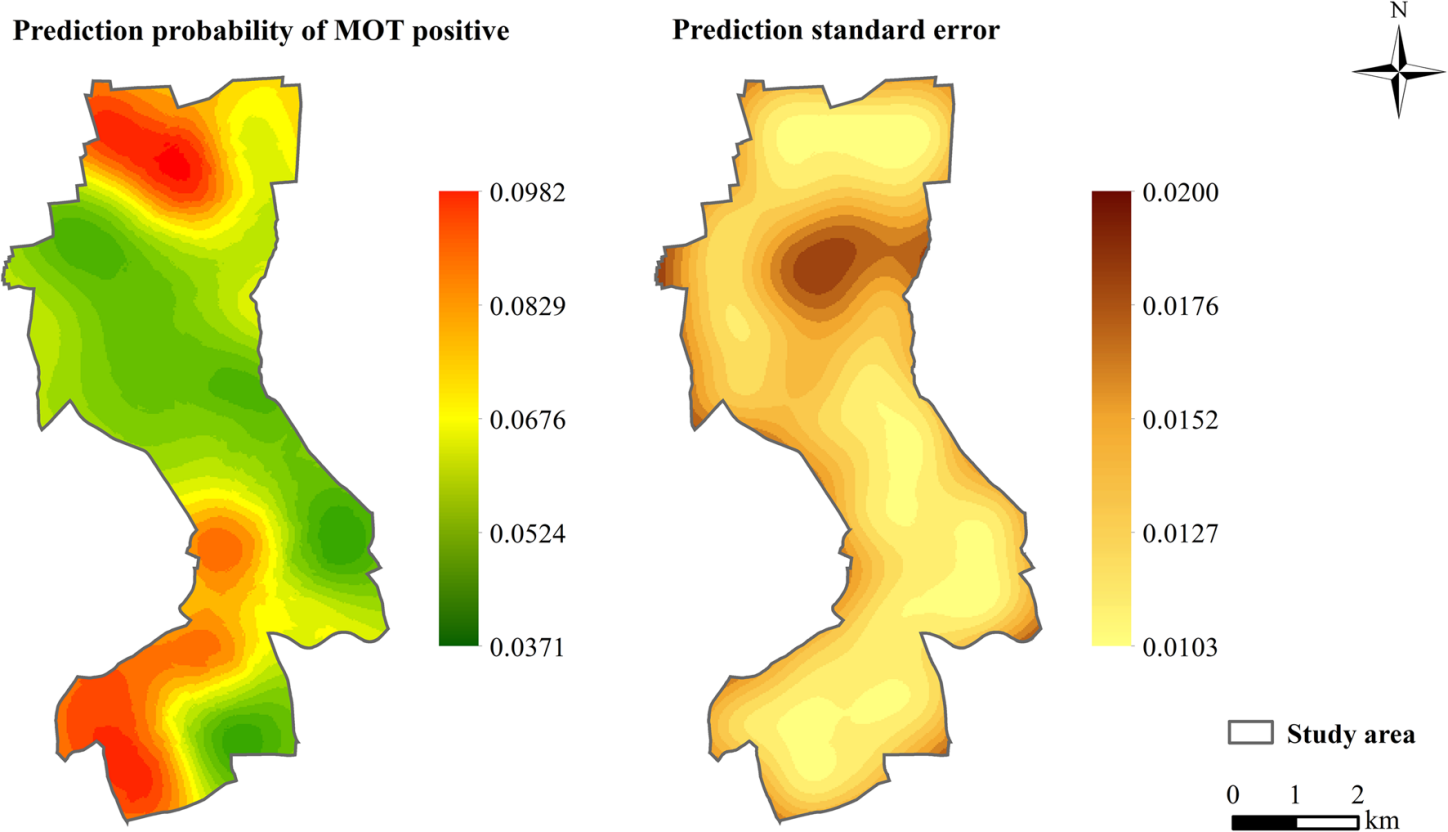
Predictions of MPI and prediction standard errors in 2018. *MPI* mosquito oviposition trap positivity index

**Fig. 8:**
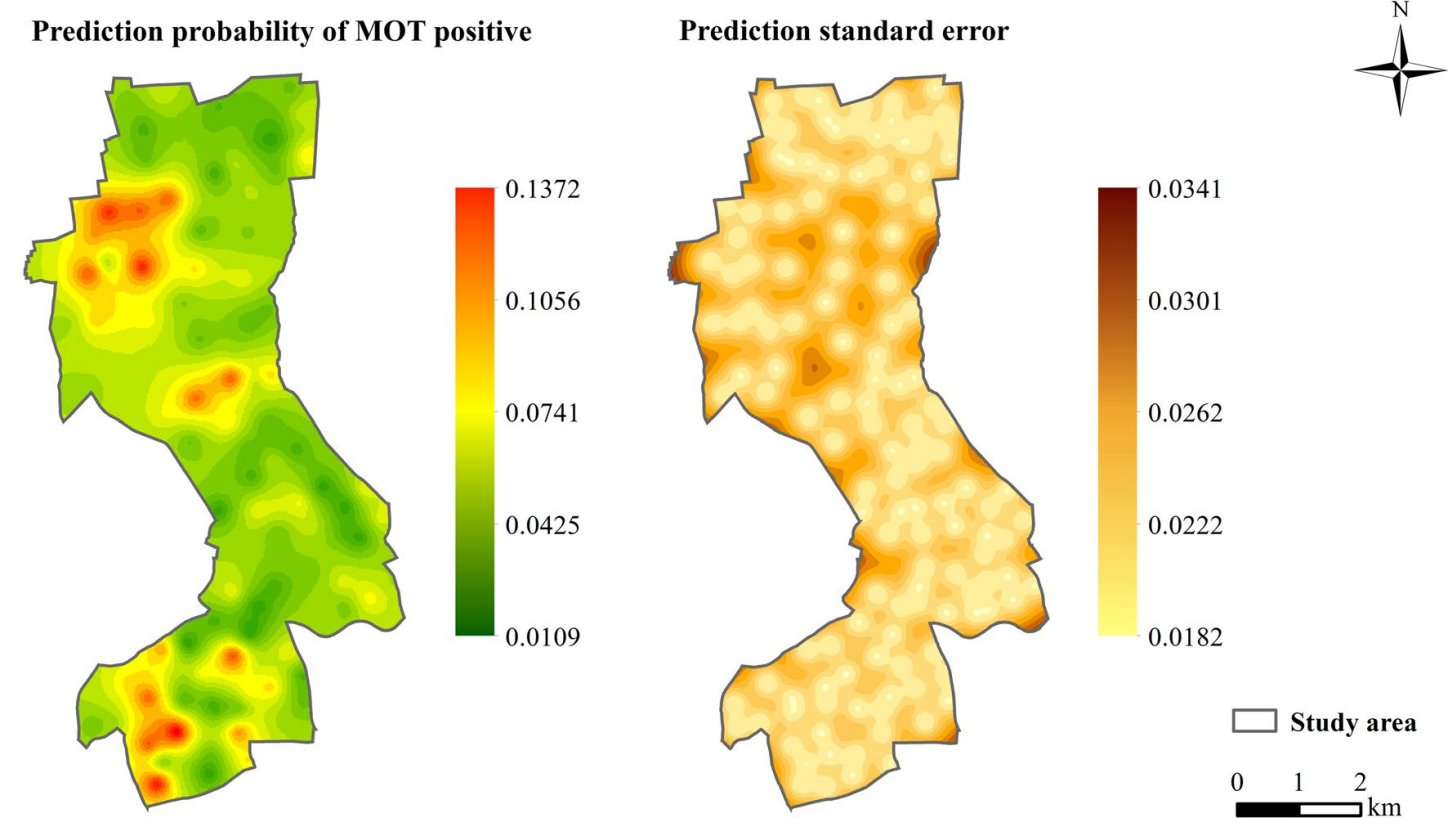
Predictions of MPI and prediction standard errors in 2019. *MPI* mosquito oviposition trap positivity index

Prediction maps associated with the standard errors based on MOT2018 (Fig. 7) and MOT2019 (Fig. 8) data showed that the highest mosquito abundance and strong spatial clustering were in the southern and northern regions of the study area in 2018, and in the southern and central areas in 2019. The prediction of standard errors quantified the degree of data uncertainty for each location. According to this analysis, the prediction error was the lowest around where MOTs were set in the study area. Overall, the leave-one-out cross-validation statistics (Table 3) with the value of RMSS approaching 1 showed that the predicted models were reliable for map production.

**Table 3:** Leave-one-out cross-validation statistics

Year	Kriging type	Range (m)	RMS	MS	RMSS	ASE
2018	Gaussian	1910	0.0336	0.0023	0.9935	0.0338
2019	Exponential	900	0.0326	− 0.0268	1.0085	0.0320

*RMS *root mean square, *MS* mean standardized, *RMSS* root mean square standardized, *ASE* average standard error

## Discussion

To the best of our knowledge, this study is the first to apply traps together with geostatistical methods to develop a routine mosquito surveillance program in China. Through this new program, a better understanding and more targeted *Ae. albopictus* control area was able to be achieved. We carried out this work in an urban area of Shanghai, where the human population density is high, with a higher blood-feeding rate of *Ae. albopictus* [35]*.* In addition, *Ae. albopictus* is the dominant mosquito species, and sometimes the sole vector, in urban areas of China [36].

According to the Implementation Program of National Vector Surveillance launched by China’s Center for Disease Control and Prevention, MOTs and LTs should be routinely applied for *Ae. albopictus* monitoring. Among the immature mosquito survey methods, MOTs have several advantages, including low cost, easy deployment, and noninvasive setup [37]. LTs have the advantage of simple operation, relatively objective monitoring results, and simultaneous monitoring at multiple sites [38]. In this program, each subdistrict, as a monitoring unit in Shanghai, has a sample size of 2 LTs and 50 MOTs in a park and a residential area, but the spatial scale is limited for evaluating *Ae. albopictus* distribution [39, 40]. To obtain a more accurate seasonal and spatial distribution of the species in order to identify areas in need of effective target control, we developed an improved scheme dividing the subdistricts into 133 units. We also evaluated the accuracy of MOTs in combination with geostatistical analysis as a practical tool for monitoring the spatial distribution of *Ae. albopictus* in an urban area of Shanghai. Compared to the original system, the improved monitoring program obtained information on a unit scale rather than a subdistrict scale, including the spatial distribution of *Ae. albopictus*, and provided a finer spatial resolution to determine the need and allocation of effective resources for control.

The MPI peaked in July in both 2018 and 2019, whereas the LT collection peaked in July in 2018 and in August in 2019. However, the indices of LT remained high from July to September in both years. Consistent with the study by Gao et al., we found a significant correlation between monthly sampling yields [41]. The *Ae. albopictus* populations in the urban areas of Shanghai slowly increased from May, peaked from July to September, and declined after September, which coincides with the seasonal high temperatures and precipitation, and is also consistent with previous reports [42, 43].

In this study, we did not observe a spatial autocorrelation for the LT collections during different periods, possibly due to the small number or low density of LTs during these periods. As the spatial analyses used distances to establish neighbors, low numbers of neighbors may have resulted in lower statistical significance. The spread of *Ae. albopictus* is limited by short-range flight, with a maximum distance of 600–800 m [44, 45]*,* which is close to the peak global Moran’s *I* of MOT. Duncombe et al. [46] suggested the mosquito traps be placed < 1200 m from each other, but in this study the maximum distance between LTs and the nearest neighbor was > 1500 m (Table 1). The spacing of LTs was too large to detect spatial autocorrelation in the sample area [47]. In our study, two LTs were arranged in each subdistrict for different areas, which contributed to the different spatial density of the traps. More LTs evenly distributed with detailed geographical information will improve current surveillance in Shanghai.

According to our results, there was a significantly positive spatial autocorrelation of MOT2018 and MOT2019 across the study area, with a maximum distance of 600 m for the peak global Moran’s *I* (Table 2). The semivariograms showed that mosquito collections from MOTs more than 1900 m in 2018 and 900 m in 2019 did not show a spatial autocorrelation (Figs. 7, 8). We focused on the spatial relationship with the risk of dengue fever, and we used the binomial areal kriging model because it is more suitable for MOT data, with the advantage of applying count data over polygons. The areal kriging model can make predictions and determine standard errors for all points within and between the input polygons by taking the sizes of the polygons into account [28, 48]. In this study, the estimated point semivariograms in Figs. 5 and 6 were different from the estimated empirical semivariograms for the polygons. Thus, the areal kriging can produce more accurate predictions than point kriging with values assigned to the polygon centroids [30, 49]. The kriging maps revealed different oviposition hot spots in 2018 versus 2019, which may be attributed to the varying mosquito abundance and seasonal distribution from year to year due to changes in temperature, precipitation, and humidity [50, 51]. In addition, the MOT2018 data may provide a reference for strict vector control in high-density areas in 2019, causing a lower MPI density at these sites compared to other regions in 2019. Combined with the binomial areal kriging model, the improved surveillance scheme obtained a more accurate spatial distribution of *Ae. Albopictus*, and identified areas in need of effective target control.

Currently, some countries use a combination of mosquito monitoring and GIS. In Brazil, Noleto constructed a map to indicate sites with the largest number of collected eggs [52]. In Singapore, the GIS monitors the network of 2000 ovitraps placed island-wide to better understand vector trends and identify hot spots and risk areas where there is a danger of high *Aedes* infestation [53]. Using the GIS, an alert system was created from a synthesis of geospatial data on ovitrap indices in Hong Kong at the district level [54]. Compared to these countries, our new monitoring project can obtain data on the distribution of mosquitoes on a smaller scale, providing a basis for accurate allocation of public health resources and targeted control of *Ae. albopictus* density.

This surveillance project can be improved by combining spatial sampling or adding climate and environmental variables affecting *Ae. albopictus* abundance, which is recommended to improve the accuracy of the spatial interpolation [55]. The spatial sampling method with the machine learning random forest algorithm for climatic variables in *Aedes* abundance prediction can optimize the distribution of monitoring traps [56]. Future studies should include the preferred Normalized Difference Vegetation Index (NDVI) and the human population in spatial modeling of abundance, which will increase the accuracy and comprehensiveness of the model [24, 25].

## Conclusions

In conclusion, an improved surveillance system with MOTs based on units can predict areas of *Ae. albopictus* abundance at non-sampling locations. This approach can improve our understanding of the spatial and temporal distribution of *Ae. albopictus* in urban regions of Shanghai and is a practical method for decision-makers to target vector control and management of mosquitoes with a finer spatial resolution. Future studies should explore the application of this monitoring program on a larger scale, and more data should be collected to validate this improved method.

## Data Availability

All relevant data are within the paper.

## Abbreviations

MOT: Mosquito oviposition trap
LT: Light trap
MPI: MOT positivity index
